# Evaluation of Morlet Wavelet Analysis for Artifact Detection in Low-Frequency Commercial Near-Infrared Spectroscopy Systems

**DOI:** 10.3390/bioengineering11010033

**Published:** 2023-12-27

**Authors:** Tobias Bergmann, Logan Froese, Alwyn Gomez, Amanjyot Singh Sainbhi, Nuray Vakitbilir, Abrar Islam, Kevin Stein, Izzy Marquez, Fiorella Amenta, Kevin Park, Younis Ibrahim, Frederick A. Zeiler

**Affiliations:** 1 Biosystems Engineering, Faculty of Engineering, University of Manitoba, Winnipeg, MB R3T 5V6, Canada; marquezi@myumanitoba.ca (I.M.); amentaf@myumanitoba.ca (F.A.); 2 Biomedical Engineering, Faculty of Engineering, University of Manitoba, Winnipeg, MB R3T 5V6, Canada; log.froese@gmail.com (L.F.); amanjyot.s.sainbhi@gmail.com (A.S.S.); vakitbir@myumanitoba.ca (N.V.); islama9@myumanitoba.ca (A.I.); steink34@myumanitoba.ca (K.S.); younis.ibrahim@umanitoba.ca (Y.I.); 3 Section of Neurosurgery, Department of Surgery, Rady Faculty of Health Sciences, University of Manitoba, Winnipeg, MB R3A 1R9, Canada; gomeza35@myumanitoba.ca; 4 Department of Human Anatomy and Cell Science, Rady Faculty of Health Sciences, University of Manitoba, Winnipeg, MB R3E 0J9, Canada; 5 Undergraduate Medicine, Rady Faculty of Health Sciences, University of Manitoba, Winnipeg, MB R3E 3P5, Canada; parkk2@myumanitoba.ca; 6 Centre on Aging, University of Manitoba, Winnipeg, MB R3T 2N2, Canada; 7 Division of Anaesthesia, Department of Medicine, Addenbrooke’s Hospital, University of Cambridge, Cambridge CB2 0QQ, UK; 8 Department of Clinical Neuroscience, Karolinska Institutet, 171 77 Stockholm, Sweden

**Keywords:** artifact management, cerebral bio-signal analysis, traumatic brain injury, hemodynamic monitoring, near-infrared spectroscopy, brain tissue oxygen saturation

## Abstract

Regional cerebral oxygen saturation (rSO_2_), a method of cerebral tissue oxygenation measurement, is recorded using non-invasive near-infrared Spectroscopy (NIRS) devices. A major limitation is that recorded signals often contain artifacts. Manually removing these artifacts is both resource and time consuming. The objective was to evaluate the applicability of using wavelet analysis as an automated method for simple signal loss artifact clearance of rSO_2_ signals obtained from commercially available devices. A retrospective observational study using existing populations (healthy control (HC), elective spinal surgery patients (SP), and traumatic brain injury patients (TBI)) was conducted. Arterial blood pressure (ABP) and rSO_2_ data were collected in all patients. Wavelet analysis was determined to be successful in removing simple signal loss artifacts using wavelet coefficients and coherence to detect signal loss artifacts in rSO_2_ signals. The removal success rates in HC, SP, and TBI populations were 100%, 99.8%, and 99.7%, respectively (though it had limited precision in determining the exact point in time). Thus, wavelet analysis may prove to be useful in a layered approach NIRS signal artifact tool utilizing higher-frequency data; however, future work is needed.

## 1. Introduction

Trajectory modeling using bio-signal analysis, which facilitates prognosis on a patient-specific level, has emerged as a beneficial method of optimizing patient care by leveraging signal analysis techniques and the understanding of pathophysiological processes [1,2,3,4,5]. This approach relies upon a continuous stream of multi-modal data recorded at the bed side [1,3,6,7]. Bedside monitoring induces various artifacts into data streams, like device disruption, patient motion, and loss of signal quality [8,9,10,11]. The manual removal of artifacts requires personnel with an understanding of the bio-signal, is time consuming, is riddled with opportunity for human error, and has an associated time lag, all of which impedes the ability of clinicians to use the data in real time [3,10]. This necessitates a semi-/fully autonomous method of artifact clearing for multi-modal high-frequency data streams [3,10,12,13,14]. Thus, work is ongoing to improve bedside artifact detection and removal in real time, with such work vital to the improvement of robust and trustworthy data analysis. Artifact detection and removal for most applications takes the form of simple methods like thresholding and gross mean value assessment. Though simple thresholding methods can be effective, their utility in removing more complicated and interspaced artifacts can be limited. More complicated waveform analysis methods, like the Pan–Thompkins method for systemic blood pressure, have been used to detect artifactual waveforms [15]. However, other high-frequency data streams are yet to have a methodology developed for the continuous assessment of good signals. Moreover, methods like the Pan–Thompkins can be limited in their ability to detect and assess artifacts that appear to behave like correct waveforms [16]. Thus, other approaches should be explored to better remove these artifacts.

Of the emerging high-frequency data streams, near-infrared spectroscopy (NIRS) is a non-invasive method of monitoring regional cerebral oxygen saturation (rSO_2_) and has gained extensive interest in the clinical setting [17,18,19,20,21]. Apart from having its own potential clinical prognostic values in varying disease states, rSO_2_ has also shown to be an effective metric for measuring changes in cerebral blood flow (CBF) [21,22,23]. The non-invasive nature of NIRS devices reduces the difficulty of implementation compared to invasive cerebral hemodynamic monitoring methods like intracranial pressure (ICP) monitoring [24]. Despite these benefits, NIRS data streams are particularly rife with artifacts due to patient motion [9,11] pads falling off, and signal distortion due to hematomas [25] or other cerebral tissue [26]. Moreover, there has yet to be an effective method developed to assess high-frequency artifact detection and removal. A solution to this problem may lie in continuous wavelet transformation (CWT) using a Morlet wavelet.

CWT has grown in popularity in cerebral physiological signal analysis as it enables the transformation of time-domain data into the time–frequency domain [18,27,28,29,30]. The temporal localization of wavelets is advantageous compared to the use of fast Fourier transformations (FFT) which only transforms time-domain data to the frequency domain [31,32,33,34]. The transformation of the rSO_2_ signal into the time–frequency domain allows for a more optimal determination of oscillatory physiological phenomena [10,32,35]. Furthermore, wavelet analysis has the benefit of being able to accomplish similar artifact detection as other methods like thresholding and waveform analysis, thus offering an all-encompassing approach to data analysis [27,29].

High-frequency oscillations (0.6–2.0 Hz) in systemic and cerebral blood flow corresponding to cardiac activity [29] can be identified using wavelet analysis in ABP (systemic blood flow) and rSO_2_ signals (cerebral blood flow). Wavelet analysis is the most robust and adaptive method for spectral signal analysis and thus is the most capable of assessing the frequency of physiological morphologies and artifacts associated with NIRS. This study will use commercially available NIRS devices (sampling frequency < 1 Hz) for the initial identification and removal of signal loss artifacts. This is critical in the development of an all-encompassing wavelet-analysis-based artifact detection method [1]. When higher-frequency data can be obtained using commercially available NIRS devices, wavelet-based simple artifact identification will be able to be applied in conjunction with wavelet-based morphological artifact identification. An all-encompassing method of artifact removal for rSO_2_ signals is considered novel to the field and will save valuable resources typically spent manually artifact clearing these signals. Moreover, this study demonstrates the main concepts and mathematics behind wavelet analysis, and in a practical example. The work here is the foundation for larger applications of CWT analysis and automatic data curation, which will build on the concepts discussed here.

## 2. Materials and Methods

### 2.1. Patient Population and Data Collection

This was a retrospective observational study using digital physiologic data collected at Health Sciences Centre, at the University of Manitoba. Three distinct populations of archived data were leveraged for this study: (A) Critically ill traumatic brain injury patients (TBI, n = 83), (B) elective spinal surgery patients (SP, n = 27), and (C) healthy control volunteers (HC, n = 103). For the TBI population, all were adult patients (>18 yrs old) with moderate/severe TBI (Glasgow Coma Scale 12 or less), requiring invasive ICP monitoring, as determined by the Brain Trauma Foundation guidelines [36]. The SP population consisted of those undergoing elective non-intradural spinal operations for degenerative pathology without any history of cerebrovascular disease. Finally, HC individuals consisted of a population of volunteers that had no history of neurologic or cardiovascular diseases. Demographic information was extracted from the database to provide a population summary of common variables found, including TBI patients’ hematomas or contusions present during the recording process. The three distinct populations were selected as they represent the key types of patients where the NIRS device is used: (A) Injured and medicated state (i.e., TBI), (B) Healthy medicated state (i.e., SP), and (C) Healthy non-injury/non-medicated state (i.e., HC).

### 2.2. Physiologic Signal Acquisition

High-frequency A-P was obtained for TBI and SP patients through arterial lines connected to pressure transducers (Baxter Healthcare Corp. CardioVascular Group, Irvine, CA, USA) zeroed at the level of the tragus in the TBI cohort and at the level of the right atrium in the SP cohort. ABP was obtained using the Finapres NOVA device (Finapres Medical Systems B.V., Enschede, The Netherlands) on each patient’s finger for the HC volunteer cohort. rSO_2_ was determined with NIRS regional oximetry of the left and right frontal lobes as the ratio of oxyhemoglobin (oxyHb) to total hemoglobin (tHb) (Covidien INVOS 5100C or 7100, https://www.medtronic.com/covidien/en-us/products/cerebral-somatic-oximetry/invos-7100-system.html accessed on 20 November 2023.

All signals were recorded using digital data transfer or digitized via an A/D converter (DT9803/DT9804/DT9826; Data Translation, Marlboro, MA, USA) and stored using the ICM+ software (Cambridge Enterprise Ltd., Cambridge, UK, http://icmplus.neurosurg.cam.ac.uk accessed on 20 November 2023. The Covidien devices have a maximum sampling rate of 1 Hz. Signal artifacts were removed using both manual and automated methods prior to further processing or analysis, identical to past work by our lab [37,38,39,40]. Each data set was examined by expert personnel who manually removed/marked the remaining artifacts in ICM+ software. This manually marked data formed the “ground-truth” comparator for our CWT method (described below) of artifact detection.

### 2.3. Signal Processing

The raw rSO_2_ and ABP were initially processed such that wavelet analysis could be properly conducted. Raw data points recorded as Not a Number (NaN) were removed completely using automated methods as wavelet analysis requires complete data streams. The ABP signal was standardized to the 1 Hz rSO_2_ sampling rate. ABP or rSO_2_ data that had been recorded as 0 but were limited to 10 data points (10 s) in length were replaced with the interpolated median value using an updating window. All signal analysis work was conducted using ICM+ software and MATLAB. Morlet CWT was used to perform a complex transformation of the time-series ABP and rSO_2_ signals into the time–frequency domain. A Morlet wavelet analysis was conducted over the rSO_2_ and ABP signals over a 10 min window that updated every minute for HC and SP cohorts. Analysis was similarly conducted on a 10 min window that updated every minute and a 60 min window that updated every 10 min for TB patients as there were more data collected from these patients. As stated in the MATLAB documentation, Morlet analysis uses an orthogonal wavelet scaling filter (low-pass finite impulse response filter), a scaling function of a symmetry parameter gamma equal to 3, and a time–bandwidth product of 60, with 10 voices per octave over a 0.002 Hz and 2 Hz (this equals 64 frequency components exponentially equally spaced) [41]. Though the full discussion on these aspects is outside of the scope of this paper, a quick summary, and practical considerations for this are as follows. A Morlet wavelet was chosen for this application due to its common use in work analyzing cerebral bio-signals. It has a more direct implementation than other wavelet methods (given the mathematics), and offers qualitative analysis to time-related changes of the multiple dimensions available in a free induction decay analysis [42] (though other methods like Daubechies should be explored). It has the added benefit of behaving like human perception, visual, and auditory discernment. Given the common implementation of CWT, the current MATLAB implementation optimizes the energy spread over the frequency components, to give a well-balanced transform for overall frequency accuracy and amplitude. This is not to say that Morlet is the best for NIRS/ABP signal analysis as other methods offer other aspects that are perhaps more important for CWT, though for practical and demonstrative purposes, Morlet is sufficient.

Morlet wavelet transformation involves convolving the time-series signal using basic functions generated from the Morlet mother wavelet [43]:

(1) $$W\left(s,t\right)=\frac{1}{\sqrt{s}}\int_{-\infty }^{\infty }\psi \left(\frac{u-t}{s}\right)g\left(u\right)\;du\;$$

where W(s,t) is a wavelet coefficient, ψ is the Morlet mother wavelet, g(u) is rSO_2_ or ABP time-series data, u denotes time, *s* is the scaling factor, and *t* is translation in time. The Morlet mother wavelet is created using a complex sine wave multiplied by a Gaussian function [43]:

(2) $$\psi \left(u\right)=\frac{1}{\sqrt[{4}]{\pi }}*e^{-i*2\pi *u}*e^{\frac{-u^{2}}{2}}\;$$

where ψ (u) similarly denotes the Morlet mother wavelet, *u* denotes the time variable, and *i* indicates an imaginary number (*i* = $\sqrt{-1}$ ). At each time instant, the time-series data are multiplied by a sine wave of different Frequencies between the defined range with a bell-shaped Gaussian at its center. Two factors impact the magnitude of wavelet coefficients in the time–frequency domain. The magnitude of change in the time-series data and the frequency of its oscillations. This is how the time-series data are transformed to the time–frequency domain. To compare the bifrontal rSO_2_ signals to ABP relationships (as a potential method to identify viable signal segments), the cross-wavelet transform was used [44]:

(3) $$W_{xy}\left(s,t\right)=W_{x}^{*}\left(s,t\right)xW_{y}\left(s,t\right)$$

The result was the cross-spectrum, which is a complex value, the local phase of which is defined as the inverse tangent of the imaginary component by the real component of the cross-spectrum [45]:

(4) $$\theta =\tan^{-1}\frac{I(W_{xy}\left(s,t\right))}{R(W_{xy}\left(s,t\right))}$$

The local phase between the two values is used to calculate semblance, which is defined as [45]:

(5) Sm=cosθ

The θ ranges from values from −π to π , and Sm ranges from −1 (inversely correlated), 0 (uncorrelated), to +1 (correlated). Semblance is a descriptor of the local phase angle between the two signals’ wavelet, with 1 meaning the waves have perfect alignment and −1 where the waves are entirely anti-aligned. Thus, semblance of 1 indicates that the change in one signal is perfectly reciprocated in another.

Coherence is defined as the square of the cross-spectrum by the normalized individual power spectra [44]:

(6) $$CO_{xy}=\frac{|S(W_{xy}\left(s,t\right)){|}^{2}}{|S(W_{x}{\left(s,t\right))|}^{2}*|S(W_{y}{\left(s,t\right))|}^{2}}$$

where * indicates the complex conjugate of the wavelet coefficient, *S* is a smoothing operator, and $CO_{xy}$ is coherence. Coherence returns a value in the exclusive range of 0 to 1, where a value close to 0 indicates very little correlation, and a value close to 1 indicates high correlation. The continuous Morlet wavelet transformation of the signals involves the mother wavelet being scaled to different frequencies [44]. Accurate NIRS and ABP data will have a coherence value within the exclusive range (0 to 1). Wavelet coherence describes how related the change in two signals is, indicating how much of the change in one signal’s wavelet is described by the other signal (similar to the traditional correlation coefficient [46]). Simple artifacts were intended to be removed, which were cases in which the signal was lost completely (rSO_2_ signal had a value of 0), which can be attributed to sensors falling off the scalp of the patient. The removal of more complex morphological artifacts that did not correspond to physiological processes was not in the scope of this paper, given limitations related to sampling frequency from commercially available NIRS systems employed in healthcare environments. The utilization of Morlet wavelets for simple artifacts is not computationally efficient when alternative methods like thresholding or FFT could be used. However, wavelet analysis has the potential to be used to identify more complex artifacts and verify signal adequacy using the presence of respiratory and cardiac oscillations. This proof-of-concept study will determine if a wavelet analysis method could be utilized to develop an algorithm capable of addressing simple artifacts in addition to more complex morphological artifacts.

### 2.4. Signal Analysis—Methods Applied to Identify Artifact Segments

The following section provides a description of how CWT of frontal lobe rSO_2_ signals and ABP signals were utilized in artifact detection of rSO_2_ signals.

#### 2.4.1. Overview of CWT Artifact Detection for rSO_2_

The transformation of time-series rSO_2_ and ABP data into the time–frequency domain using wavelet decomposition facilitated the analysis of one hemisphere’s rSO_2_ signal and its relationship with ABP when undergoing slow-wave oscillations. The wavelet coefficients for rSO_2_ as well as the semblance and coherence between the rSO_2_ and ABP were plotted in the time–frequency domain for the raw, uncleaned rSO_2_ data for HC, SP, and TBI patients. HC and SP patients were analyzed in 10 min windows updating every minute. TBI patients were analyzed in less detailed 60 min windows updating every 10 min. Since TBI data sets had smaller oscillations and longer recording times, a larger 60 min window was used to properly capture the oscillations. The resultant plots for the absolute wavelet coefficients, coherence, and semblance results were examined for a characteristic result that could be used to indicate a signal artifact. Characteristic results were categorized in frequency bands corresponding to literature results for slow-wave cardiovascular oscillation frequencies [29,47,48]. Frequency band I had a range of approximately 0.5–2 Hz with a peak at 1 Hz corresponding to cardiac activity [29,47,49]. Frequency band II was defined between 0.15 and 0.5 Hz and is corresponding to respiratory activity [29,47]. Frequency band III was defined between 0.05 and 0.15 Hz, corresponding to myogenic vascular activity [27,29]. Frequency band IV exists between 0.02 and 0.05 Hz, corresponding to neurogenic activity [27,29]. Frequency bands V and VI exist between 0.0095 and 0.02 Hz and 0.005 and 0.0095 Hz, respectively, which correspond to two endothelial responses [27,29,50]. Frequencies were limited in MATLAB by the sampling frequency of the commercially available NIRS device used (1 Hz).The highest frequency for coherence was 0.3683 Hz. The highest possible wavelet frequencies were used as they captured more rapid changes in the time-domain data sets. To highlight this limitation of commercial systems, the data of a single volunteer were sampled using the non-commercially available Artinis OxyMon NIRS device that used a sampling rate of 250 Hz. This allowed for coherence, semblance, and wavelet coefficients to be determined for frequency bands I–VI.

#### 2.4.2. Absolute Wavelet Coefficients Applied in Artifact Clearance for rSO_2_ Across Populations

Simple signal loss artifacts common in rSO_2_ signals are occurrences where motion, disconnection, or transducer failures will result in rSO_2_ having a rapid change in mean value between consecutive samples. The absolute value of wavelet coefficients from raw rSO_2_ data were expected to be able to identify the beginning and the end of artifacts. A signal loss involving a large change in rSO_2_ magnitude was expected to cause a spike in the magnitude of the absolute value of wavelet coefficients and be dramatically larger than those in normal oscillating data. The absolute value of wavelet coefficients for rSO_2_ evaluated in the time–frequency domain and the time-domain rSO_2_ signal were compared. The average wavelet coefficient magnitude across all frequencies and the corresponding rSO_2_ magnitude was obtained for each timestamp. The nature of wavelet coefficients made it impossible to utilize their magnitude to differentiate cases in which the rSO_2_ signal was lost and immediately regained or was lost for an extended period and regained. Wavelet coherence and semblance were evaluated for their ability to differentiate these two cases.

#### 2.4.3. Wavelet Coherence between ABP and rSO_2_ Applied in Artifact Clearance for rSO_2_ Across Populations

Throughout the duration of the loss of rSO_2_ signals, there were no oscillations. As such, the coherence between rSO_2_ and ABP was expected to be either 0 or 1. A coherence value of 0 indicated that the oscillating ABP signal had no correlation or similarity to rSO_2_ when signal had been lost. A value of 1 was used to indicate when the signal had been lost simultaneously in ABP and rSO_2_. The results of wavelet coherence between rSO_2_ and ABP in the time–frequency domain in different frequency bands were compared to the corresponding time-domain signals for each time stamp. This analysis provided useful information into the differentiation between lost or active rSO_2_ signals.

#### 2.4.4. Wavelet Semblance between ABP and rSO_2_ Applied in Artifact Clearance for rSO_2_ Across Populations

Wavelet semblance was like coherence in that, when the rSO_2_ signal was lost, it was expected that semblance resulted in an erroneous value. The nature of semblance calculating the local phase angle between two signals (ABP and rSO_2_) would result in an artifact when the rSO_2_ signal was lost for an extended period. Contrary to coherence, there was no expected result in response to the rSO_2_ signal being lost, as it was an artifact. Semblance was more difficult to use than coherence in this application; as a result, the evaluation was not included in the manuscript. More details are included in Appendix C.

#### 2.4.5. Proposed Method of CWT Artifact Clearance for rSO_2_

To utilize wavelet analysis to clear raw rSO_2_ signals of simple signal loss artifacts, the following procedure was proposed:

1. Observationally establish thresholds for absolute value of wavelet coefficients of rSO_2_ to indicate when signal is lost or regained;
2. Observationally establish thresholds in frequency bands to indicate when the rSO_2_ signal is not oscillating (lost for a prolonged period).

The accuracy of the proposed method to identify all artifacts in the raw rSO_2_ data were determined using two criteria. The first was comparing the number of artifacts determined through manual inspection of the raw rSO_2_ data to the number of these artifacts that were detected using the proposed method (thresholds in wavelet coefficients or coherence). The second was to understand false positives, which were the number of points erroneously removed by the algorithm compared to the manually cleaned rSO_2_ data. This was done by comparing the number of points that had a coefficient result above the defined threshold to the number of points that this threshold had been used to remove. The proposed method was not expected to perform differently between different patient populations (HC, SP, and TBI); however, its efficacy was validated by testing the ability of the proposed method to remove artifacts in each population. The proposed method was also sub-tested on two patient factors: cerebral autoregulation (CA) state and cerebral injury pattern for additional validation of robustness.

#### 2.4.6. Sub-Group Analyses—Cerebral Autoregulation State

Impaired CA is common in TBI patients [51,52]; as such, the versatility of the artifact identification method was contingent upon its functionality in patients with impaired and intact CA, which necessitated sub-group analyses for CA state. Cerebral oxygenation index (CO_x__a) was used as a metric to quantify cerebral autoregulatory health, it was calculated using a continuous moving Pearson correlation of 30 consecutive and paired 10 s averaged values of ABP and rSO_2_ that updated every minute [51,53]. Observationally, it was determined that CO_x__a < 0 indicated intact CA, and CO_x__a > 0.3 indicated grossly impaired CA [51,54,55,56]. These were based on tissue oxygenation index (TO_x_) thresholds for intact and impaired CA and CO_x__a thresholds corresponding to individuals with impaired CA attributed to hypertension [51,56]. TO_x_ values exceeding 0.3 are known to affect the upper and lower limits of the cerebral autoregulatory ability to maintain cerebral blood flow [56]. CO_x__a values exceeding 0.36 were linked with a loss of autoregulation related to hypertension [51]. To determine the impact of CA health on the success of artifact removal, the artifact removal success rates were calculated for HC, SP, and TBI patient data sets that had been filtered for intact CA using CO_x__a for SP and TBI data sets that had been filtered for impaired CA on an individual data point basis.

#### 2.4.7. Sub-Group Analyses—Cerebral Injury Patterns

The presence of a hematoma or contusion was expected to have an impact on the quality of the rSO_2_ signal measured using the commercially available device [25,57]. Blood pooling underneath the NIRS pads would have distorted the infrared light reaching the cerebral vasculature. The presence of a hematoma is common in TBI cases [58], which necessitates that the artifact identification method function despite a patient having one. The success rates of the method were compared for TBI cases with the presence or absence of a hematoma below the NIRS pad. The impact of hematomas and contusions on the rate of success for artifact identification was analyzed by comparing the success rate and percent of data points that were artifacts for cases with and without hematomas or contusions (as manually identified on computed tomographic neuroimaging).

To further evaluate the effect of hematomas or contusions on rSO_2_ signals, the wavelet method between manually identified patients with or without hematomas was evaluated. Pearson correlation coefficients were calculated between the two frontal lobe hemispheres’ rSO_2_ signals, with the resulting Pearson coefficients used to identify if there was a significant difference between the “normal” and “abnormal” states. This method had a limited effect in determining artifacts and as such, has been removed from the main manuscript (for more details, refer to Appendix H).

## 3. Results

**Figure 1:**
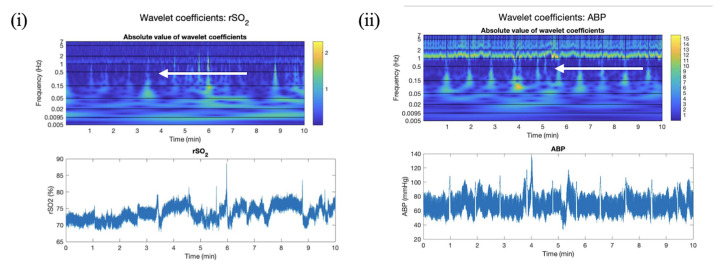
Wavelet coefficients for model signal (250 Hz). Time–frequency domain color plots of wavelet coefficients and time-series data in patient data sampled at 250 Hz (measurements per second) for (**i**) rSO_2_ and (**ii**) ABP with arrows indicating a 1 Hz peak, where rSO_2_ is cerebral oxygen saturation, and ABP is arterial blood pressure measured in millimeters of mercury (mmHg).

The small amount of data from the OxyMon Mk III system (Artinis Medical Systems, Elst, The Netherlands) was utilized briefly to highlight limitations associated with CWT analysis in commercial systems. The OxyMon NIRS data were sampled at 250 Hz. The single patient recorded using a non-commercially available NIRS device was a male 29 years old, with no personal history of cardiovascular or neurovascular issues. A model signal was identified using this patient’s rSO_2_ and ABP data sampled at 250 Hz. The results for the wavelet coefficients for ABP and rSO_2_ signals are depicted in Figure 1. There are notably higher wavelet coefficients present around a frequency of 1 Hz that are present in both ABP and rSO_2_ results (which correspond to the cardiac mechanisms).

**Figure 2:**
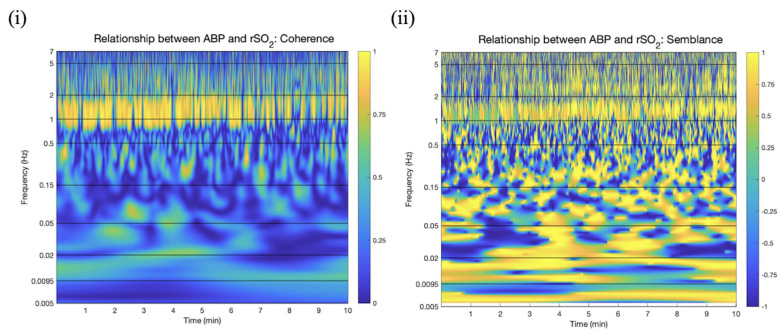
Coherence and semblance between ABP and rSO_2_ (250 Hz). Time−frequency domain color plots of wavelet (**i**) coherence and (**ii**) semblance between rSO2 and ABP in patient data sampled at 250 Hz (measurements per second), where rSO_2_ is cerebral oxygen saturation, ABP is arterial blood pressure is measured in millimeters of mercury (mmHg), and frequency is measured in Hz (cycles per second).

High wavelet coefficients in this region for both signals led to a high coherence between the two signals in this band. Semblance is close to 1, which corresponds to a direct correlation between the two signals. The coherence and semblance between these two signals are depicted in Figure 2. This infers that there is a direct relationship between ABP and rSO_2_ corresponding to cardiac activity (0.5–2 Hz). This characteristic band of high coherence and semblance could be used to define a valid rSO_2_ signal on a patient-by-patient basis.

The limitations of the sampling rate for commercially available NIRS devices make it impossible to measure oscillations corresponding to cardiac activity using wavelet analysis. However, a simple signal loss artifact clearance method was devised using data sampled using a commercially available NIRS. This wavelet-analysis-based method can be used in conjunction with a wavelet-analysis-based method to remove morphological artifacts.

The TBI patient population demographic can be found in Table 1. The SP (n = 27) and HC (n = 103) population demographics are included in Appendix A and Appendix B, respectively. For the TBI population, there were 83 patients, with 57 (79.2%) being males and a median age of 43.5 years.

### 3.1. Absolute Wavelet Coefficients Applied in Artifact Clearance for rSO_2_ Across Populations

**Figure 3:**
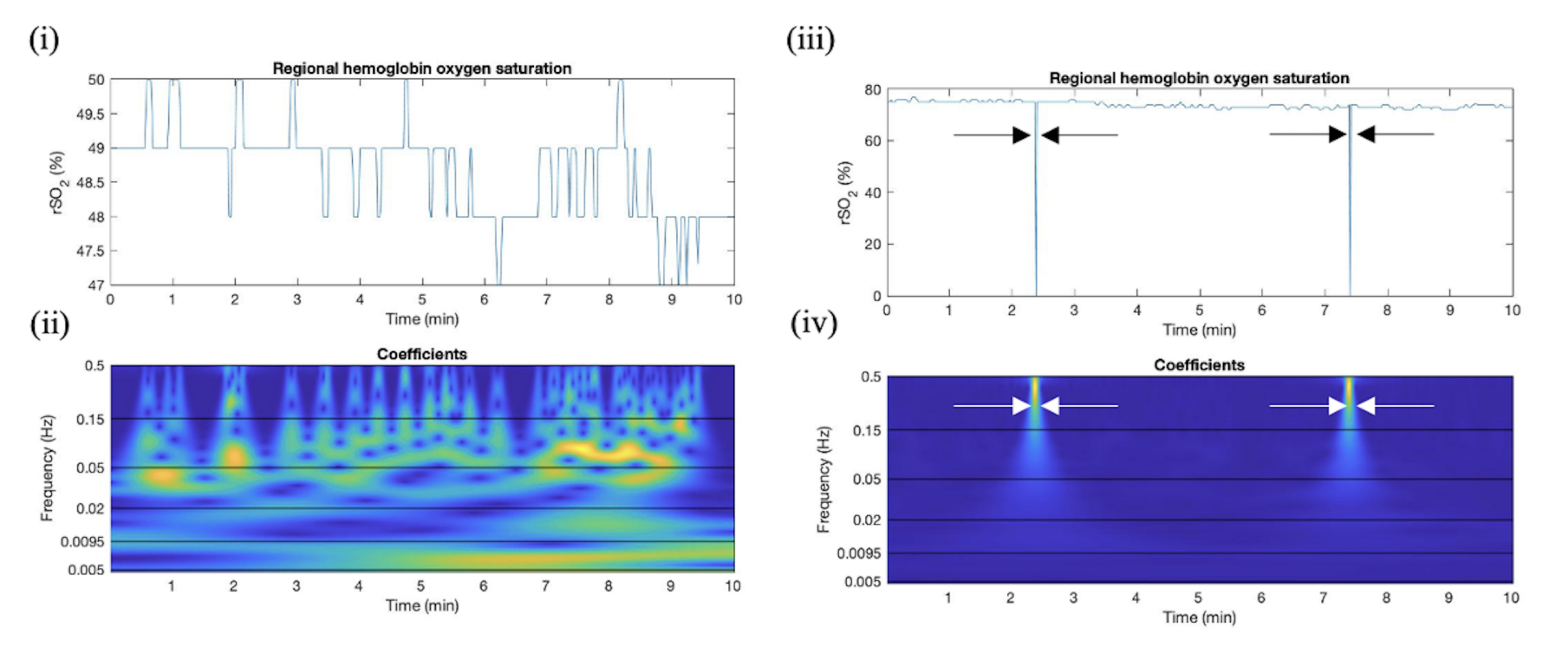
Results for absolute value of wavelet coefficients when rSO_2_ signal is valid and artifactual. This plot depicts (**i**) uncleaned time-series rSO_2_ plots recorded on right frontal lobe of SP patient (there is a lower magnitude in the rSO_2_ signal, which could be attributed to the sedative present during elective spinal surgery or the skin color of the patient). (**ii**) Corresponding wavelet coefficient time–frequency series color plot, where frequency is measured in Hz (oscillations per second) blue indicates absolute wavelet coefficient values below 1 (wavelets do not oscillations in signal well). (**iii**) uncleaned time-series rSO_2_ plots recorded on right frontal lobe of HC patient where signal loss artifacts are present. (**iv**) Corresponding wavelet coherence time–frequency series color plot. Arrows indicate the location of the artifacts in time where yellow indicates +1 (high correlation) and blue indicates 0 (low correlation).

As expected, there was a distinct peak in the magnitude of the absolute value of the rSO_2_ wavelet coefficients in the time–frequency domain plots, which corresponded to rSO_2_ signal losses. The rSO_2_ time-domain data and the corresponding absolute value of wavelet coefficients for an HC data set are included in Figure 3. This figure depicts the magnitude of wavelet coefficients when the signal is maintained and when it is briefly lost.

Displayed in Figure 3 is a peak in absolute wavelet coefficient and a peak in high coherence corresponding to the signal loss in both rSO_2_. This demonstrates the peak in coherence that occurs when both signals are lost, which can be used to identify artifacts. Data were sampled at 1 Hz (measurements per second), where rSO_2_ is cerebral oxygen saturation.

### 3.2. Wavelet Coherence between ABP and rSO_2_ Applied in Artifact Clearance for rSO_2_ Across Populations

**Figure 4:**
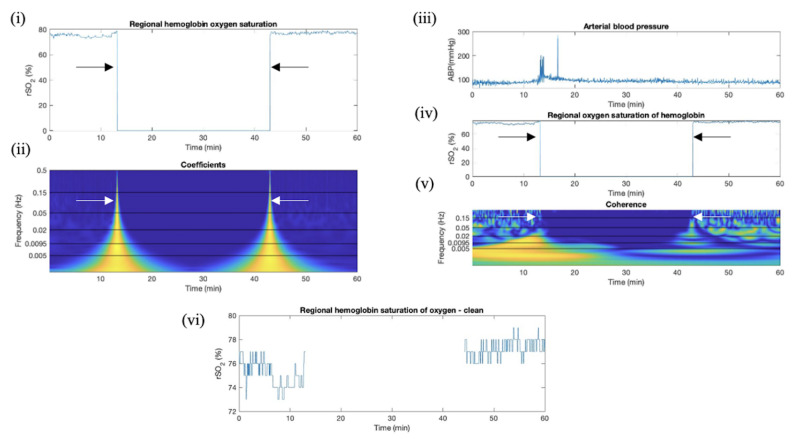
Wavelet coefficient and coherence artifact identification in TBI rSO_2_ data, data section removal using rSO_2_ and ABP. This plot depicts (**i**,**iv**) uncleaned time-series rSO_2_ plots. (**ii**) Corresponding wavelet coefficient time–frequency series color plot, where frequency is measured in Hz (oscillations per second) blue indicates absolute wavelet coefficient values below 1 (wavelets do not oscillations in signal well). (**iii**) Time-series arterial blood pressure plot. (**v**) corresponding wavelet coherence time–frequency series color plot where yellow indicates +1 (high correlation) and blue indicates 0 (low correlation). (**vi**) Cleaned time-series rSO_2_ plot. Arrows indicate the location of the artifacts; the start is indicated by wavelet coefficient magnitude and the section where signal was lost is indicated by coherence. The ABP signal was still oscillating, as a result, the coherence appeared as 0 when the rSO_2_ signal was lost. Data were sampled at 1 Hz (measurements per second), where rSO_2_ is cerebral oxygen saturation.

Coherence was useful in determining whether a peak in wavelet coefficient magnitude was a solitary spike in rSO_2_ or a longer signal loss artifact for which the entire segment had to be removed. The loss of the rSO_2_ signal resulted in no oscillations, making the wavelet coefficient value near 0. The resulting coherence between rSO_2_ and ABP signals was depicted as either 0 or 1. This was used to indicate the presence of a signal loss artifact. An exemplar plot is included in Figure 4.

There were also cases in which the signal was lost and regained rapidly. The resultant plots for wavelet coefficients and coherence are depicted in Appendix D, Appendix E and Appendix F.

### 3.3. Proposed Method of CWT Artifact Clearance for rSO_2_

The magnitude of the absolute wavelet coefficients was observationally determined to be larger when corresponding to an artifact due to the dramatic change in rSO_2_ magnitude. The average wavelet coefficient magnitude across the frequency bands (0.0048 Hz to 0.5 Hz) for which wavelet coefficient analysis was conducted was calculated. It was determined that if the average absolute wavelet coefficient for a time instant exceeded 4 in SP and HC populations and 3.5 in TBI populations, it indicated a spike in magnitude indicative of an artifact. A shortcoming of using solely absolute wavelet coefficient magnitude was that a continued signal loss in the adjacent data points to this spike could not be removed as there was no significant magnitude change. To determine whether the adjacent points corresponded to a signal loss or typical oscillations, the magnitude of coherence was used. It was not possible for the coherence between ABP and rSO_2_ to be perfectly correlated ( $CO_{xy}$ = 1) or uncorrelated ( $CO_{xy}$ = 0). Thus, the coherence result being either 0 or 1 throughout the upper frequency band (0.2067 to 0.3683 Hz) indicated that the rSO_2_ signal was lost. Absolute wavelet coefficients and coherence at higher frequencies captured more rapid changes in the signal. A rapid rSO_2_ signal loss, while ABP continued to oscillate, was observed in coherence results by having an average result close to 0. In cases when the rSO_2_ and ABP signals were lost simultaneously, the average result was close to 1. An average coherence result outside the 0.15 to 0.7 range was determined to indicate the potential of a signal loss. There were other artifact types that were not removed using this filter. As such, it was determined that the coherence was close to 0 in frequency bands 0.0046 to 0.0205 Hz or 0.2067 to 0.3683 Hz when these artifacts occurred. An average coherence result below 0.15 in either of these bands indicated the presence of an artifact. Coherence filters were sequentially applied to data points adjacent to artifacts, to indicate whether the signal was immediately regained or if it extended for multiple seconds. A summary of the results of this method of artifact identification when it was applied is included in Table 2. The performance of CWT absolute wavelet coefficients and coherence values at detecting artifacts are compared to the manually annotated data. A main limitation of using thresholding in the absolute value of wavelet coefficients to remove artifacts is that non-artifactual points close in proximity to artifacts are erroneously removed. This is also included in Table 2.

All the data points across the populations were analyzed. Artifacts in the rSO_2_ signal were identified as those with a magnitude below 1. Thresholding in the absolute wavelet coefficient with a magnitude above 4 and 3.5 in HC and SP/TBI populations, respectively. Thresholding in coherence was used in three frequency bands (coherence outside of 0.15 and 0.7 in the frequency band between 0.2067 and 0.3683 Hz, below 0.15 in the frequency band between 0.0046 and 0.0205 Hz, and below 0.15 in the frequency band between 0.0517 and 0.1548 Hz) to determine artifacts. The error rate is the percentage of erroneously removed data points over all the data points analyzed (i.e., CWT-identified artifact that was not manually identified as such). There are additional depictions of how this method was applied to remove artifacts in HC, SP, and TBI data sets. They are included in Appendix D, Appendix E, and Appendix F, respectively.

### 3.4. Sub-Group Analyses—Cerebral Autoregulation State

CA health did not seem to affect the success rate of the proposed methods. The success rates for HC, SP, and TBI patient data sets that were filtered for data points with intact CA (CO_x__a < 0) were 100%, 99.8%, and 100%, respectively. The success rate for SP and TBI patient data sets that were filtered for data points with impaired CA (CO_x__a > 0.3) were 99.6% and 98.6%, respectively. A more detailed summary of the results of using the proposed method for varying CA states is included in Appendix G.

### 3.5. Sub-Group Analyses—Cerebral Injury Patterns

The presence of hematomas and contusions did not seem to significantly affect the success rate of the proposed method. A summary of the results of using the artifact identification method in the presence and absence of a hematoma on the side analyzed are included in Table 3. Also included is the influence of hematomas and contusions on the number of artifacts during recording.

Hematomas/contusions were identified in the ICU only if they were present below the NIRS pad. Artifacts in the rSO_2_ signal were identified as those with a magnitude below 1. Thresholding in the absolute wavelet coefficient with a magnitude above 4 and 3.5 in HC and SP/TBI populations, respectively. Thresholding in coherence was used in three frequency bands (coherence outside of 0.15 and 0.7 in the frequency band between 0.2067 and 0.3683 Hz, below 0.15 in the frequency band between 0.0046 and 0.0205 Hz, and below 0.15 in the frequency band between 0.0.0517 and 0.1548 Hz) to determine artifacts. Percent artifacts is the number of artifactual points to the number of points recorded throughout the session (with or without hematoma). It should be noted that Table 3 depicts a significant difference in the number of artifacts in the recorded data with/without hematomas or contusions, with such an injury pattern significantly increasing the number of artifacts in the data set.

## 4. Discussion

Wavelet analysis of rSO_2_ data sampled using a non-commercially available NIRS device was able to identify oscillations in the signal due to cardiac activity. The presence of these oscillations may be leveraged to indicate morphological artifacts in the rSO_2_ signal. However, limitations in the sampling rate of commercially available NIRS devices make it impossible to assess these oscillations. Wavelet analysis was successfully used as a method of artifact clearance for data sampled at 1 Hz from a commercially available NIRS device in HC, SP, and TBI data sets. Thus, demonstrating that CWT can be used in data monitoring for artifact detection in NIRS, marking the first step for autonomous data management.

**Figure A8:**
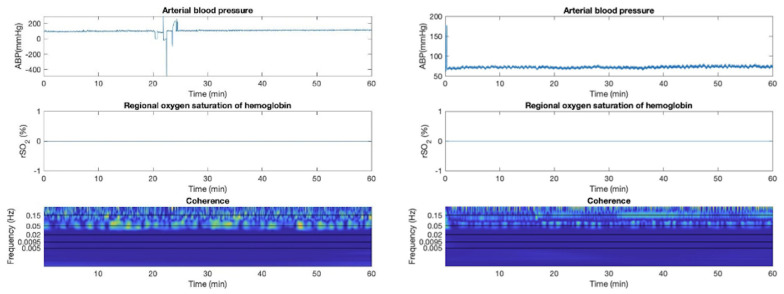
Time –series data and coherence for long-signal-loss artifact. This plot depicts the artifact in coherence that appeared in response to the signal being lost for an entire window in TBI patients where yellow indicates +1 (high correlation) and blue indicates 0 (low correlation). Due to the nature of wavelet coherence determining the correlation between the wavelet coefficients of the two signals, the ABP signal being momentarily stationary appears as having some coherence. This is not present when both signals are oscillating. This artifact was removed using filter that below 0.15 in frequency band between 0.0046 and 0.0205 Hz.

**Figure A9:**
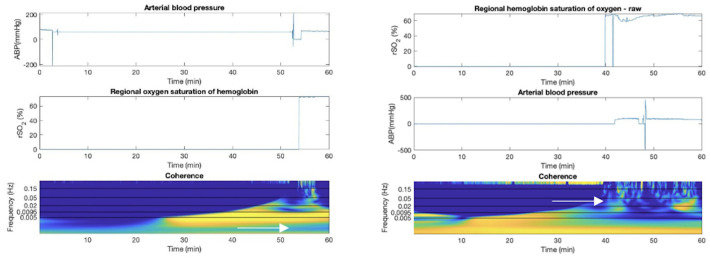
Time –series data and coherence depicting erroneous coherence artifact where yellow indicates +1 (high correlation) and blue indicates 0 (low correlation). This plot has arrows, which indicate artifacts that are difficult to remove using coherence thresholding. Signal losses in rSO2 and signal losses in ABP that did not coincide at the same time instant resulted in this artifact type. Removed using filter that below 0.15 in frequency band between 0.0517 and 0.1548 Hz indicated artifact.

The average absolute wavelet coefficients of 4 and 3.5 were observationally determined to indicate the presence of a signal loss artifact in HC/SP and TBI populations, respectively. A large change in rSO_2_ magnitude resulted in a proportionate spike in absolute wavelet coefficient magnitude. A smaller threshold was selected for TBI data as the normal magnitude of rSO_2_ was smaller in this population due to inconsistent baseline cerebral tissue oxygenation that puts patients at risk of secondary injuries [59]. When a spike in wavelet coefficient magnitude was detected, filters in wavelet coherence were used to evaluate whether the signal was lost and quickly regained or was lost for an extended period. A coherence threshold of 0.15 to 0.7 between 0.2067 and 0.3683 Hz (band II) was successful in removing these artifacts in both HC and SP populations. However, two artifact types in TBI populations proved more challenging to remove when the rSO_2_ signal was lost for an extended period in TBI data sets. Long-signal-loss artifacts, which occurred in TBI data and are depicted in Appendix F (Figure A8), were a challenge to remove using wavelet thresholding. This artifact was a prolonged signal loss in which rSO_2_ was lost for the entire duration of a window. The low sampling rate and accuracy of commercially available NIRS devices resulted in the ABP signal being measured as having the same value for several seconds. Due to the nature of wavelet analysis, when the rSO_2_ signal was lost, and ABP was measured as briefly not oscillating, which resulted in some coherence being prevalent in band II, and as a result was not captured by the threshold in that band. This artifact likely appeared only in TBI populations due to a weaker ABP and rSO_2_ signal because of lower systemic and cerebral blood flow as the patient was under sedation, and that data were being collected in the ICU where the commercial NIRS devices may not have had a proper connection throughout recording. An additional filter was applied to encapsulate this artifact. A threshold of coherence under 0.15 between 0.0046 and 0.0205 Hz (band IV and V) denoted an artifact. A second artifact type that was difficult to remove was erroneous coherence, which is depicted in Appendix F (Figure A9). This artifact occurred when ABP and rSO_2_ were not lost simultaneously. This resulted in coherence being present in bands II, VI, and V. An additional filter of coherence under 0.15 between 0.0517 and 0.1548 Hz removed many of these artifacts. In continuous wavelet transformations, temporally localized wavelets are multiplied by each data point [31,43,44]. This presents a main shortcoming of using wavelet thresholding in artifact clearance. A spike in wavelet coefficient magnitude corresponding to a signal loss will also increase the magnitude of the coefficients of the adjacent points. The magnitude of thresholds selected needed to encapsulate each signal loss artifact while mitigating the adjacent non-artifactual points (false positives) that were erroneously removed as is depicted in Table 3. The number of points erroneously removed for HC data sets was particularly notable as it exceeded the number of artifacts removed. This method is no more advantageous than basic thresholding in removing simple signal loss artifacts; however, this method can be applied in conjunction with a wavelet analysis method capable of detecting morphological artifacts for a proficient all-encompassing wavelet-based method of artifact clearing rSO_2_ signals. In sub-group analyses of TBI patients with hematomas and contusions, it was determined that their presence beneath the NIRS pad did not significantly affect the ability to detect a signal loss or success rate of artifact identification using wavelet analysis. The presence of a hematoma did not affect the ability to detect the loss of a signal. Hematomas and contusions did seem to make rSO_2_ signals more prone to artifacts depicted by the ratio of artifactual points to points recorded within the sub-group. In the absence of a ‘good’ rSO_2_ signal as a comparison, a Pearson correlation between frontal lobe rSO_2_ signals was used. Indicated in the Results with additional information in Appendix H, the presence of a hematoma or contusion did not significantly impact the Pearson correlation between the rSO_2_ signals. Data sampled at a higher frequency and accuracy could be analyzed to better understand the effect of hematomas on the magnitude and oscillations of rSO_2_ signals. This work validated wavelet artifact detection for thresholding, and some minor preliminary analysis on higher-frequency waveform analysis. Within this, there is great promise with the universal applicability of the wavelet methodology for various types of artifact detection. CWT has the advantage over other simpler methods as it can be used to extract oscillatory phenomena and is more dynamic to physiological patterns. Furthermore, CWT as a method holds many unique aspects associated with physiological morphologic detection and overall system robustness, it will inevitably be used in some fashion in physiological artifact management. As such this work is a practical demonstration of the foundational aspects of CWT, a simplistic demonstration of the math and how it can be used for physiological data assessment. This is a preliminary step in developing a wavelet-analysis-based artifact clearance method capable of removing all artifacts from rSO_2_ signals. An automated method of removing artifacts from this bio-signal is novel to the field.

### Limitations and Future Directions

The wavelet coefficient and coherence artifact removal technique were solely evaluated on TBI, SP, and HC data sets that were sampled at 1 Hz. It was not able to be tested on data sampled at 250 Hz using non-commercially available NIRS technology as there were no signal loss artifacts present in the data sampled. Additionally, wavelet thresholding was exclusively used to remove simple signal loss artifacts. As such, the integration of such methods using more complex datasets, leveraging these methods with other artifact clearing methodologies was not explored and could lead to further improvement of the error detection. The impact of sampling frequency was demonstrated using a single healthy volunteer with NIRS data sampled at 250 Hz using a research-grade system. Future analysis of rSO_2_ waveforms would significantly benefit from higher-frequency data from different patient groups as the higher sampling rate and accuracy would accommodate wavelet analysis to be conducted on higher-frequency bands. Oscillations in rSO_2_ and ABP would be able to be analyzed with more wavelet frequencies, providing an easier distinction between physiological oscillations and artifacts using absolute wavelet coefficients and coherence. There was a distinct band of higher absolute wavelet coefficient, high coherence, and semblance indicating correlation in the 1 Hz range, which corresponded to cardiac activity. Coherence and semblance depict a direct relationship in frequency band I (0.5–2 Hz). This has the potential to be leveraged to define a valid rSO_2_ signal in future work but could not be evaluated using this data set as the healthy individual’s data included no artifacts. Higher-frequency data also have the potential to offer an improved understanding of baseline rSO_2_ signals. The low sampling rate in commercially available NIRS devices made it impossible to identify the oscillations in blood flow corresponding to physiological processes in the signal. A higher sampling frequency would enable ranges of the absolute value of wavelet coefficients for varying cerebral vascular health and hematomas to be defined. The issue of identifying long-signal-loss artifacts in TBI populations also would likely be absent using a higher sampling rate. The wavelet coherence and semblance between the rSO_2_ and ABP have the potential to be used in the diagnosis of CA impairment. It is also necessary to conduct analysis using the methods defined in this paper to validate that it is suitable as a method of artifact clearance in higher-frequency data and whether it can be improved. In addition, wavelet analysis must be evaluated for its ability to identify different types of errors that may be present in higher-frequency data. If the data were collected in a controlled environment, it would also be worthwhile to manufacture artifacts by intentionally creating signal losses in data sets.

## 5. Conclusions

Wavelet coefficients and coherence were able to be leveraged to autonomously detect artifacts from rSO_2_ signals in TBI patient, SP, and HC populations. However, the erratic signal recorded using a commercially available NIRS device made it impossible to devise a method to remove more complex signal artifacts. Sub-group analyses of the presence of hematomas and contusions as well as CA health of the patient were found to not have a significant impact on the success of the artifact clearance method. Hematomas and contusions did appear to affect the frequency of artifacts. This work demonstrates the initial steps for an automated NIRS artifact management system, CWT as a method performs similarly to simpler methods with the added benefit of being more attuned to oscillatory phenomena. Future research should be conducted in the development of a more robust algorithm for higher-frequency recording data that utilizes neural networks integrating multiple signal analysis techniques to adequately clear the varying types of artifacts that occur during bedside monitoring. 

## Figures and Tables

**Table 1:** Patient demographics—TBI cohort.

Demographics	Median (Interquartile Range) or Number of Patients
Age	42 (27.5–58.6)
Sex (% Male)	68 (81.9%)
Best Admission GCS—Total	6 (4–8)
Best Admission GCS—Motor	4 (2–5)
Number with Hypoxia episode	26 (31.3%)
Number with Hypotension episode	9 (10.8%)
Number with Traumatic SAH	80 (96.4%)
Pupils	
Bilateral Unreactive	11 (13.3%)
Unilateral Unreactive	18 (21.7%)
Bilateral Reactive	52 (62.7%)
Admission Marshall CT	
V	41 (48.4%)
IV	15 (18.1%)
III	24 (28.9%)
II	3 (3.6%)
Mean ICP (mmHg)	7.9 (4.2–10.7)
% Time ICP > 20 mmHg	0.3 (0.0–2.0)
% Time ICP > 22 mmHg	0.1 (0.0–1.2)
Mean CPP (mmHg)	76 (71.3–83.5)
% Time CPP > 70 mmHg	74 (52–84)
% Time CPP < 60 mmHg	4 (0.4–6.3)
Mean rSO ${\;\;}_{2}$ (au)	68.5 (61.3–76.5)
Mean CO ${\;\;}_{x}$ _a	0.06 (0.01–0.13)
% Time CO_x__a > 0	54 (49–61)
% time CO_x__a > 0.25	22 (16–27)

where Au = arbitrary units, CO_x__a = cerebral oximetry index, CPP = cerebral perfusion pressure, CT = computerized tomography, GCS = Glasgow coma scale, ICP = intracranial pressure, mmHg = millimeters of mercury; PRx = pressure reactivity index, rSO ${\;\;}_{2}$ = regional cerebral oxygen saturation, SAH = subarachnoid hemorrhage.

**Table 2:** Summary of effectiveness of wavelet analysis in artifact identification.

Data Set	Data Points Analyzed	Artifact Points Identified (rSO_2_ < 1)	Removed Using abs Wavelet Coefficient	Removed Using Coherence	Success Rate	Erroneously Removed Points	Error Rate
HC (Right)	154,961	522	517	5	100%	3433	2.22%
HC (Left)	154,961	522	521	1	100%	3566	2.30%
SP (Right)	279,446	15,927	3143	12,756	99.8%	4645	1.66%
SP (Left)	279,446	16,175	2988	13,165	97.9%	5045	1.81%
TBI (Right)	25,557,085	4,719,300	190,505	4,513,148	99.9%	311,656	1.22%
TBI (Left)	25,557,085	5,084,529	129,516	4,945,166	99.8%	219,791	0.86%

**Table 3:** Effect of hematomas and contusions on effectiveness of wavelet analysis in artifact identification and on frequency of artifacts.

Data Set	Hematoma/Contusion	Data Points Analyzed	Artifact Points Identified (rSO_2_ < 1)	Removed Using abs Wavelet Coefficient	Removed Using Coherence	Success Rate	Percent Time Recorded Signal Was Artifact
TBI (Right)	Present	5,683,219	1,939,540	61,056	1,878,141	100%	34.1%
TBI (Right)	Absent	19,873,866	2,779,760	129,449	2,635,007	99.4%	14.0%
TBI (Left)	Present	3,982,816	2,496,637	6122	2,490,401	100%	62.7%
TBI (Left)	Absent	21,574,268	2,587,892	123,034	2,454,765	99.6%	12.0%

**Figure A1:**
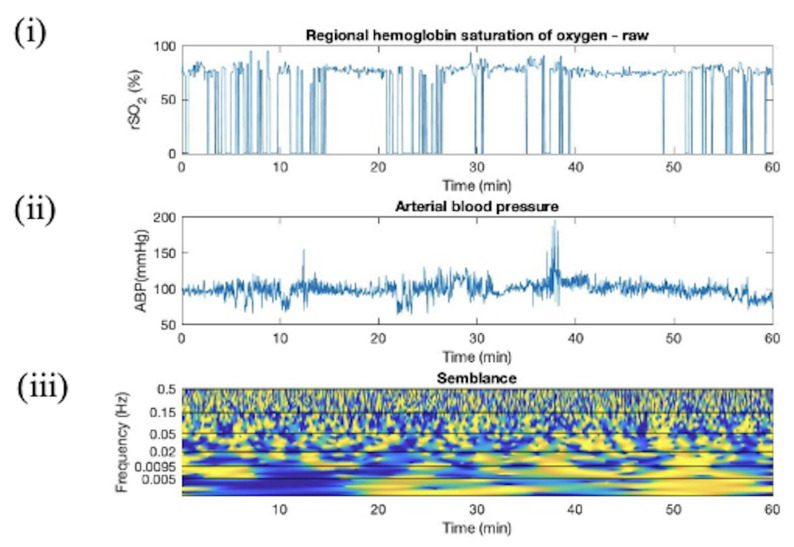
Semblance between left rSO_2_ signal and ABP signal for TBI patient data with short artifacts. This figure depicts raw TBI time-series data for (**i**) rSO_2_ and (**ii**) ABP, as well as (**iii**) the semblance between them depicted as a time–frequency color plot using a 60 min window. It depicts that semblance serves no utility for semblance in identifying these short artifacts. Yellow indicates a value of +1 (correlated), cyan indicates a value of 0 (uncorrelated), and blue indicates a value of −1 (inversely correlated).

**Figure A2:**
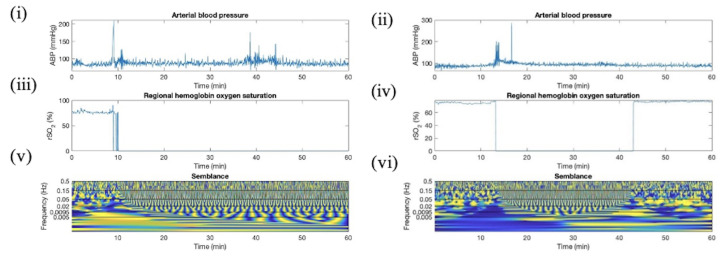
Semblance between right rSO_2_ signal and ABP signal for TBI patient data with longer artifacts. This figure depicts raw TBI time-series data for (**i**,**ii**) ABP and (**iii**,**iv**) rSO2, as well as (**v**,**vi**) the semblance between them depicted as a time–frequency color plot using a 60 min window. It depicts that semblance serves no utility for semblance in identifying artifacts when the rSO_2_ signal is lost for an extended period. This would have made it difficult to define thresholds in semblance to remove them. Yellow indicates a value of +1 (correlated), cyan indicates a value of 0 (uncorrelated), and blue indicates a value of −1 (inversely correlated).

**Figure A3:**
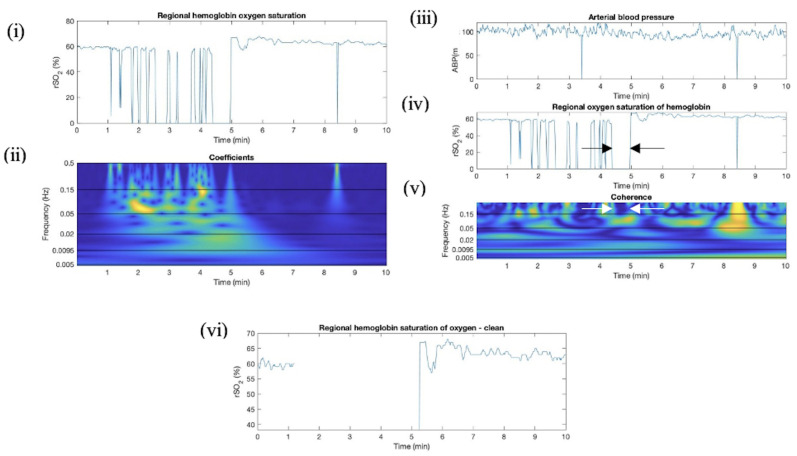
This plot depicts (**i**,**iv**) uncleaned time-series rSO_2_ data, (**ii**) a time-frequency domain color plot of absolute wavelet coefficients, (**iii**) uncleaned time-series ABP data, (**v**) time-frequency domain color plot of wavelet coefficients, and (**vi**) manually cleaned time-series rSO_2_ data. Arrows depict the segment in which coherence in the upper frequency band is used to remove the artifact. Other artifacts were removed using absolute wavelet coefficient thresholds. Blue indicates absolute wavelet coefficient values below 1 (wavelets do not oscillations in signal well).

**Figure A4:**
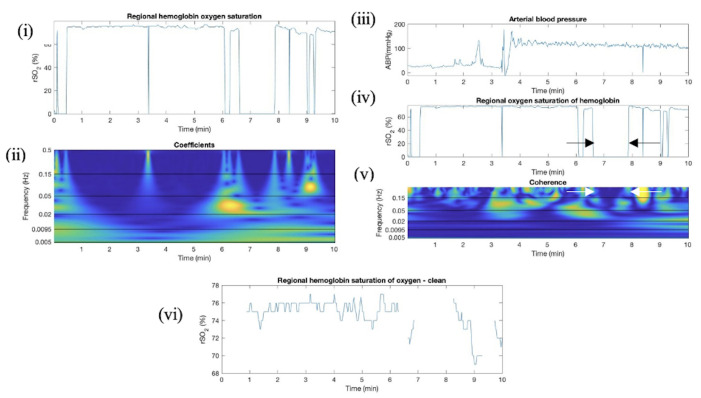
Wavelet This plot depicts (**i**,**iv**) uncleaned time-series rSO_2_ data, (**ii**) a time-frequency domain color plot of absolute wavelet coefficients, (**iii**) uncleaned time-series ABP data, (**v**) time-frequency domain color plot of wavelet coefficients, and (**vi**) manually cleaned time-series rSO_2_ data. Arrows depict the segment in which coherence in the upper frequency band is used to remove the artifact. Other artifacts were removed using absolute wavelet coefficient thresholds. Blue indicates absolute wavelet coefficient values below 1 (wavelets do not oscillations in signal well).

**Figure A5:**
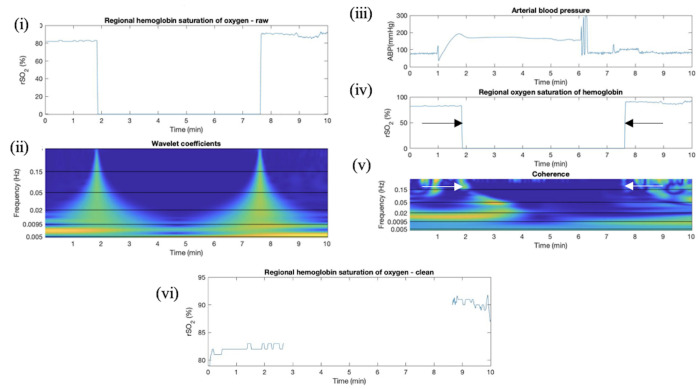
Wavelet coefficients used in artifact clearing left rSO_2_ signal for raw SP data. Right rSO_2_ signals in time domain and color plot of coefficients in time–frequency domain. This plot depicts (**i**,**iv**) uncleaned time-series rSO_2_ data, (**ii**) a time–frequency domain color plot of absolute wavelet coefficients where blue indicates absolute wavelet coefficient values below 1 (wavelets do not oscillations in signal well), (**iii**) uncleaned time-series ABP data, (**v**) time–frequency domain color plot of wavelet coherence between ABP and rSO_2_, where yellow indicates +1 (high correlation) and blue indicates 0 (low correlation), and (**vi**) manually cleaned time-series rSO_2_ data. Arrows depict the segment in which coherence in the upper frequency band is used to remove the artifact.

**Figure A6:**
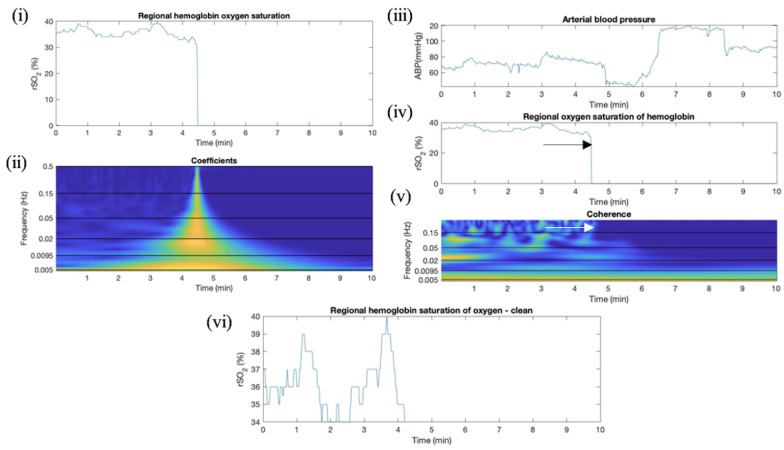
Wavelet coefficients right rSO_2_ signal for raw SP data. Right rSO_2_ signals in time domain and color plot of coefficients in time–frequency domain. This plot depicts (**i**,**iv**) uncleaned time-series rSO_2_ data, (**ii**) a time–frequency domain color plot of absolute wavelet coefficients where blue indicates absolute wavelet coefficient values below 1 (wavelets do not oscillations in signal well), (**iii**) uncleaned time-series ABP data, (**v**) time–frequency domain color plot of wavelet coherence between ABP and rSO_2_, where yellow indicates +1 (high correlation) and blue indicates 0 (low correlation), and (**vi**) manually cleaned time-series rSO_2_ data. Arrows depict the segment in which coherence in the upper frequency band is used to remove the artifact.

**Figure A7:**
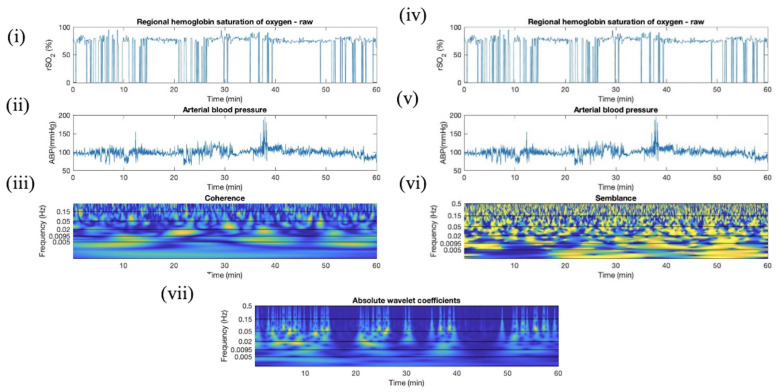
Wavelet coefficient favored compared to coherence and semblance in detecting short artifacts in right TBI rSO_2_ data. This plot depicts (**i**,**iv**) uncleaned time-series rSO_2_ plots, (**ii**,**v**) uncleaned ABP time-series data), (**iii**) coherence between ABP and rSO_2_ where yellow indicates +1 (high correlation) and blue indicates 0 (low correlation), (**vi**) semblance between ABP and rSO_2_ where yellow indicates a value of +1 (correlated), cyan indicates a value of 0 (uncorrelated), and blue indicates a value of −1 (inversely correlated), and (**vii**) absolute wavelet coefficients for rSO_2_ where blue indicates absolute wavelet coefficient values below 1 (wavelets do not oscillations in signal well). There are several rapid artifacts present in the rSO_2_ for this window. These artifacts are not indicated in coherence or semblance but are prevalent in the wavelet coefficients. Time-series rSO_2_ data indicate several losses in signal throughout the window.

## Data Availability

The data that support the findings of this article are not publicly available as they pertain to private patient information. They can be requested from the author at frederick.zeiler@umanitoba.ca.

## Abbreviations

The following abbreviations are used in this manuscript:

TBI	Traumatic brain injury
SP	Elective spinal surgery patients
HC	Healthy controls
CO_x_	Cerebral oxygenation index
TO_x_	Tissue oxygenation index

## Appendix A. Demographics from Elective Spinal Surgery Population

**Table A1:** Demographics from Elective Spinal Surgery Population.

Demographics	Median (Interquartile Range) or Number of Patients
Number of Patients	27
Male Sex	22 (81.5%)
Age	57 (52–65.5)
Weight (kg)	84.6 (69.2–100)
O2 Inhalation (%)	68 (49–100)
End-Tidal CO_2_	35 (18–37)
Arterial CO_2_	44 (37–47)
Arterial O_2_	240 (211–303)
Procedure Type	
ACDF	22 (81.5%)
Male Sex	6 (22%)
PCDF	14 (52%)
ACDF and PCDF	3 (9%)
Cervical Incision and Drainage	1 (3%)
Corpectomy	1 (3%)
Laminectomy	1 (3%)
Thoracic Decompression	1 (3%)
Duration of Recording (min)	185 (166–203)
rSO_2__L (%)	66 (59–73)
rSO_2__R (%)	67 (60–73)
MAP (mmHg)	83 (79–86)
CO_x__a_L (au)	0.17 (0.08–0.25)
CO_x__a_R (au)	0.2 (0.1–0.28)
% time CO_x__a_L > +0.3 (% time)	40 (31–46)
% time CO_x__a_R > +0.3 (% time)	41 (34–48)

where ACDF = anterior cervical discectomy and fusion, au = arbitrary units, COx_a = cerebral oximetry index, kg = kilogram, MAP = mean arterial blood pressure, min = minutes, mmHg = millimeters of mercury, PCDF = posterior cervical discectomy and fusion, rSO_2_ = regional oxygen saturation.

## Appendix B. Demographics from Healthy Control Population

**Table A2:** Demographics from Healthy Control Population.

Demographics	Median (Interquartile Range) or Number of Patients
Number of Patients	103
Male Sex	43 (41.7%)
Age	26 (22–31)
Hand Dominance (Right)	89 (87%)
Duration of Recording (min)	33 (29–36)
rSO_2__L (%)	73 (69–79)
rSO_2__R (%)	72 (66–79)
MAP (mmHg)	101 (86–114)
CO_x__a_L (au)	0.14 (0.01–0.22)
CO_x__a_R (au)	0.12 (0.03–0.22)
% time CO_x__a_L > +0.3 (% time)	27 (13–37)
% time CO_x__a_R > +0.3 (% time)	25 (15–38)

Au = arbitrary units, CO_x_ _a = cerebral oximetry index, kg = kilogram, MAP = mean arterial blood pressure, min = minutes, mmHg = millimeters of mercury, rSO2 = regional oxygen saturation.

## Appendix G. Sub-Group Analyses—Cerebral Autoregulation State

**Table A3:** Summary of effectiveness of wavelet analysis in artifact identification when data are filtered by CA health.

Data Set	CA Health	Data Points Analyzed	Artifact Points Identified (rSO_2_ < 1)	Removed Using abs Wavelet Coefficient	Removed Using Coherence	Success Rate
HC	Intact	64,916	511	511	0	100%
SP	Intact	558,892	32,102	6131	25,921	99.8%
SP	Impaired	208,448	5713	1470	4218	99.6%
TBI	Intact	15,556,279	247,624	147,620	100,314	100%
TBI	Impaired	9,495,501	101,894	37,703	62,802	98.6%

TBI and SP data were filtered by CO_x__a values for entire windows that were identified as having intact CA (CO_x__a < 0) and impaired CA (CO_x__a > 0.3), and HC windows identified as having intact CA (CO_x__a < 0) all using 10 min windows updating every minute. Artifacts in the rSO_2_ signal were identified as those with a magnitude below 1. Thresholding in the absolute wavelet coefficient with a magnitude above 4 and 3.5 in HC and SP/TBI populations, respectively. Thresholding in coherence was used in three frequency bands (coherence outside of 0.15 and 0.7 in the frequency band between 0.2067 and 0.3683 Hz, below 0.15 in the frequency band between 0.0046 and 0.0205 Hz, and below 0.15 in the frequency band between 0.0.0517 and 0.1548 Hz) to determine artifacts.

## Appendix H. Pearson Correlation between rSO2 Signals

The results for the Pearson correlation between rSO_2_ right and left side signals are depicted in Table A4. The table indicates the percentage of windows for which the Pearson correlation exceeded 0.2 and 0.5, respectively, as well as the number of windows iterated across the data set (60 min window for TBI, 10 min window for SP and HC). Filtering the data for artifacts and for impaired or intact CA was intended to improve the results for the Pearson correlation between rSO_2_ signals. The similarity between the rSO_2_ signal is a method of quantifying the confidence in the signals. A higher Pearson correlation instills higher confidence in the validity of the rSO_2_ signal. It can be noted in the table the effect of cleaning the data for signal loss artifacts and filtering by CA health. The presence of signal loss artifacts clearly lowers the percentage of windows in which the Pearson correlation is strong. There is a distinctly higher percentage of windows with a high Pearson correlation when the data have been filtered by CO_x__a values. It can be concluded that the criterion that the data set must satisfy using CO_x__a filtering requires a strong rSO_2_ signal.

**Table A4:** Pearson correlation results for data sets.

Data Set	*p* > 0.2 (%)	*p* > 0.5 (%)	Total Windows
TBI data	57.76	45.36	43,936
SP data	82.67	63.36	4760
TBI cleaned	85.94	67.35	26,919
SP cleaned	87.18	64.48	3885
HC cleaned	91.68	68.85	793
HC (CO_x__a < 0)	100	97.22	36
SP (CO_x__a > 0.3)	98.63	87.67	219
SP (CO_x__a < 0)	99.29	86.43	140
TBI (CO_x__a > 0.3)	100	97.53	481
TBI (CO_x__a < 0)	94.47	85.43	199

To further observe the effects of hematomas or contusions on rSO_2_ signals, Pearson correlation coefficients were calculated between frontal lobe rSO_2_ signals, comparing “normal” to “abnormal” In the TBI patient population, the presence of hematomas and contusions on either or both lobes were noted. The Pearson correlation between frontal lobe rSO_2_ signals was calculated for each window for raw TBI, HC, and SP data. The analysis was then reconducted on the cleaned data. It was also conducted on windows that had been filtered for CA impairment in TBI and SP data, and windows of HC data that had been filtered for intact CA. The percentage of windows that had Pearson values exceeding 0.2 and 0.5 were determined for each data set to quantify the results for each. The Pearson coefficient values for the relationship between frontal lobe rSO_2_ signals of 0.2 and 0.5 indicated the percent of windows in which there was a “low positive correlation” and a “moderate positive correlation”, respectively [60]. It was expected that in cases in which TBI patients had hematomas or contusions present, the Pearson correlation would be significantly lower than those without, due to blood pooling distorting the signal on that side and lack of signal variance. The effect of hematomas or contusions on rSO_2_ and its relationship to ABP was not analyzed. The data for TBI patients that had no hematoma or contusion below the NIRS pad had 86.92% and 69.10% of Pearson correlations (P) with values of *p* > 0.2 and *p* > 0.5, respectively. The cleaned TBI data that was specific to those with hematomas/contusions identified had 82.76% and 61.36% of windows with Pearson correlations with *p* > 0.2 and *p* > 0.5, respectively. These results did not differ significantly from HC and SP. The calculation of Pearson correlation for each window did not provide any additional insight into the effects of hematomas/contusions on rSO_2_ signal quality.
